# Protective effects of endurance exercise on skeletal muscle remodeling against doxorubicin-induced myotoxicity in mice

**DOI:** 10.20463/pan.2020.0010

**Published:** 2020-06-30

**Authors:** Insu Kwon

**Affiliations:** 1 Research Institute of Sports Science and Industry, Hanyang University, Seoul Republic of Korea

**Keywords:** Doxorubicin, Myotoxicity, Endurance exercise, Skeletal muscle regeneration, Satellite cell activation, Proteolytic systems

## Abstract

**[Purpose]:**

Doxorubicin (DOX) is a potent anti-cancer drug that appears to have severe myotoxicity due to accumulation. The skeletal muscle has a regeneration capacity through satellite cell activation when exposed to extracellular stimulus or damage. Endurance exercise (EXE) is a therapeutic strategy that improves pathological features and contributes to muscle homeostasis. Thus, this study investigated the effect of EXE training in mitigating chronic DOX-induced myotoxicity.

**[Methods]:**

Male C57BL/6J mice were housed and allowed to acclimatize with free access to food and water. All the mice were randomly divided into four groups: sedentary control (CON, n=9), exercise training (EXE, n=9), doxorubicin treatment (DOX, n=9), doxorubicin treatment and exercise training (DOX+EXE, n=9) groups. The animals were intraperitoneally injected with 5 mg/kg/week of DOX treatment for 4 weeks, and EXE training was initiated for treadmill adaptation for 1 week and then performed for 4 weeks. Both sides of the soleus (SOL) muscle tissues were dissected and weighed after 24 hours of the last training sessions.

**[Results]:**

DOX chemotherapy induced an abnormal myofiber’s phenotype and transition of myosin heavy chain (MHC) isoforms. The paired box 7 (PAX7) and myoblast determination protein 1 (MYOD) protein levels were triggered by DOX, while no alterations were shown for the myogenin (MYOG). DOX remarkably impaired the a-actinin (ACTN) protein, but the EXE training seems to repair it. DOX-induced myotoxicity stimulated the expression of the forkhead box O3 (FOXO3a) protein, which was accurately controlled and adjusted by the EXE training. However, the FOXO3a-mediated downstream markers were not associated with DOX and EXE.

**[Conclusion]:**

EXE postconditioning provides protective effects against chronic DOX-induced myotoxicity, and should be recommended to alleviate cancer chemotherapy-induced late-onset myotoxicity.

## INTRODUCTION

Doxorubicin (DOX) is a potent chemotherapeutic agent for various cancer types, but has accompanied side effects, including fatigue and skeletal muscle weakness, leading to limitations in cancer chemotherapy^1,2^. It also causes severe myotoxicity and muscle atrophy due to bioaccumulation^3,4^. Nuclear DNA damage^5,6^, direct or indirect oxidative stress^7,8^, mitochondrial dysfunctions (impairment of respiratory capacity and redox imbalance due to DOX high affinity for the inner mitochondrial membrane [IMM])^9-13^, and metabolic impairments^14-17^ have been suggested as the underlying molecular mechanisms for DOX-induced myotoxicity. Additionally, FOXO3α-mediated ubiquitin-proteasome system (UPS) and autophagy have been proposed as the cause of the proteolytic systems for DOX-induced myotoxicity^18-20^. Notably, DOX-induced myotoxicity often presents in a dose-dependent manner, and the degree of dysfunction occurs predominantly in the soleus (SOL) muscle than in the extensor digitorum longus^21,22^.

The human body is made up of 40% skeletal muscle; it is one of the major tissues made up of multinucleated myofibers that possess the contractile machinery to generate force and physical activity. It has plasticity, which is the ability to alter its structural and functional properties in response to exercise^23-25^. Moreover, it has resident muscle stem cells, namely, satellite cells that are required for normal muscle growth, maintenance, self-repair, and regeneration of the skeletal muscle^26-28^. Since the satellite cells are anatomically located between the sarcolemma and basal lamina of the myofibers, the proximity to myofibers suggests that they may be associated with muscle regeneration^28,29^. Although the satellite cells are quiescent under normal conditions, they are quickly activated when damage occurs^30^. Satellite cell activation results in the proliferation of myoblasts and differentiation into myotubes that are finally merged into preexisting myofibers or become new myofibers. Hence, some of the identified markers are expressed in each step with the recruitment of a progenitor and the myogenic regulatory factors (MRFs): paired box7 (PAX7), myoblast determination protein 1 (MYOD), and myogenin (MYOG)^29,31^. Previous studies reported that DOX treatment inhibits MYOD expression in vitro^32,33^. Nevertheless, whether chronic DOX-mediated myotoxicity is associated with a decrease in muscle regeneration resulting from the inactivation of the satellite cells in vivo is unknown.

Endurance exercise (EXE) training is a non-pharmacological remedy to the skeletal muscle metabolic adaptations and homeostasis maintenance in health and disease^34^. Primarily, it promotes healthy myofibers by improving mitochondrial biogenesis, enhancing oxidative capacity, and shifting the muscle-fiber type from glycolytic to oxidative traits^35-37^. Furthermore, EXE-induced autophagy is required as a compensatory mechanism for protection in neutralizing DOX toxicity^18^. It is correctly outlined for the autophagy signaling pathways involved in the health benefits of the skeletal muscle during exercise^38^. Although previous studies applied EXE preconditioning as a counteracting strategy against DOX-mediated myotoxicity and dysfunctions^18-20,39-46^, the skeletal muscle regeneration capacity with chronic chemotherapeutic DOX treatment and post-conditioning of EXE training is yet to be examined.

Therefore, this study was conducted to investigate the effect of post-conditioning EXE training against chronic DOX chemotherapy-induced myotoxicity by assessing skeletal muscle regeneration capacity.

## METHODS

### Animals

All procedures were conducted according to the principles of the Institute of Animal Care and Use Committee of University of West Florida (Approval No. 2017-005). Thirty-six 8-week-old male C57BL/6J mice were purchased from ENVIGO company (Tampa, FL, USA), and housed in a temperature (22˚C) and humidity(55%)-controlled room under a 12:12 hours light/dark cycle with food and water ad libitum. After 1 week of acclimatization, the animals were randomly divided into four groups: sedentary control (CON, n=9), endurance exercise training (EXE, n=9), doxorubicin treatment (DOX, n=9), and doxorubicin treatment and following the endurance exercise training (DOX+EXE, n=9). The body weight of all the mice was measured weekly and recorded until the end of the experiments.

### Doxorubicin chemotherapy

Doxorubicin hydrochloride was purchased from Sigma Aldrich (D1515, MO, USA). Mice assigned to the DOX chemotherapy groups were intraperitoneally injected with DOX (5 mg/kg/week) for 4 weeks, while the non-DOX chemotherapy groups were injected with an equivalent volume of phosphate-buffered saline (PBS) as a vehicle. A dosage of DOX administration was obtained from previous studies^12,41,43^.

### Endurance exercise training

**Figure 1.**
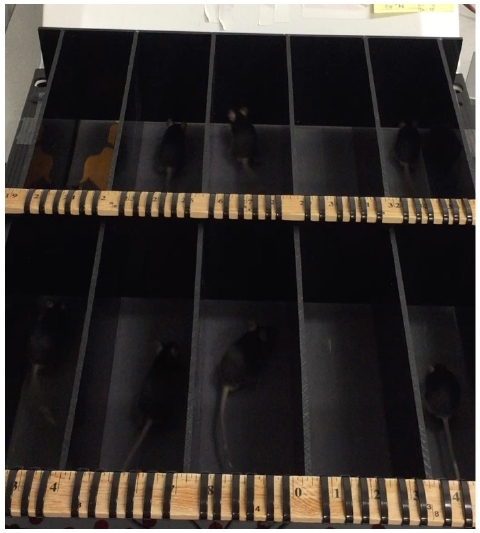
Motorized mice treadmill. A treadmill consists of a conveyor belt, individual track, and a modulator to control the speed and time of running.

After administration of DOX, mice assigned to EXE training groups were familiarized with endurance running for 30 min daily consecutively for 5 days. A motorized mice treadmill was used at a speed of 8~12 m/min (Fig. 1). After acclimation, the mice performed endurance exercise (rate of 12~15 m/min) for 60 min daily for 4 weeks. To exclude possible confounding factors resulting from environmental stresses (e.g., treadmill noise and stay in treadmill) rather than exercise effects, the mice assigned to the non-exercise training group were placed in the same training room during the entire training periods. The intensity of this exercise (70-80 % of VO_2max_) was determined from a previous study^47^. The use of electrical shock was avoided to prevent unfavorable stress for the trained mice; instead, bristle brushes were used at the end of each lane, and all the animals successfully performed the training.

### Tissue collection

All animals were sacrificed by cervical dislocation, and the SOL muscles from both legs were immediately dissected 24 hours after the last session of the endurance exercise training and weighed. The left SOL muscle was immediately frozen in liquid nitrogen and stored at -80˚C for western blot assay. The right side SOL muscle was mounted with optimal cutting temperature (OCT) freezing medium, rapidly frozen in isopentane pre-cooled with liquid nitrogen, and stored at -80˚C for histological analysis.

### Hematoxylin and eosin (H&E) staining

Following sacrifice and dissection, 10 μm of the SOL muscles were cut using a cryostat (Leica CM-1860) and mounted on microscope slides. The slides with the tissue sections were incubated in hematoxylin solution (#6765001, ThermoFisher Scientific) and washed in tap water sequentially for 5 min. Thereafter, the slides were consecutively incubated in 1% HCL (#A144S-500, Fisher Scientific) and 70% ethanol for 1 min, then wash in tap water for 5 min. After that, the slides were incubated in an eosin solution (#HT110332-1L, Sigma-Aldrich) for 2 min. H&E-stained tissue sections were successively immersed in 70%, 80%, and 90% ethanol for 1 min, after which the sections were incubated in 100% xylene to ethanol ratio (1:1) for 1 min, and then dipped into 100% xylene (#X3F-1GAL, Fisher Scientific) for 3 min. Mounting was performed using the VECTASHIELD^®^ Mounting Medium (#H-1400, Vector Laboratories). Finally, the slides were visualized under a microscope (40X) with EVOS^®^ FL Auto Imaging System (#AMAFD1000, Life Technologies™).

### Myosin heavy chain (MHC) isoforms expression

The slides with SOL muscle tissue sections (10 μm) were air-dried for 30 min at room temperature (RT) and fixed with 4% diluted formaldehyde solution (#F8775, Sigma-Aldrich) for 20 min on ice. For permeability, the slides were dipped into jars in 0.5% triton X-100 (#T8787, Sigma-Aldrich) with 1X PBS (pH=7.4) for 30 min at RT and then washed (3×5 min) with 1X PBS. Further, the sectioned slides were incubated in a blocking solution, 5% normal goat-serum (#50062Z, ThermoFisher Scientific), for 1 hour at RT. Subsequently, the slides were incubated with the designated primary antibodies (dystrophin: #PA1-21011, ThermoFisher Scientific; type I MHC: #BA-D5, and type IIa MHC: #SC-71, Developmental Studies Hybridoma Bank) for 24 hours at 4˚C. Next, the incubated slides were washed (3×5 min) with 1X PBS and incubated with the corresponding secondary antibodies (Alexa 555^®^: #A-21429 for dystrophin, Alexa 350^®^: #A-21140 for type I MHC, and Alexa 488^®^: #A-21121 for type IIa MHC, ThermoFisher Scientific) for 1 hour at RT. Then, the dying tissue-sectioned slides were washed (3×5 min) with 1X PBS, and mounted with mounting medium (H-1400, Vector Laboratories). Finally, the tissue sections were visualized using EVOS^®^ FL Auto Imaging System (#AMAFD1000, Life Technologies™) using a 40X objective. The composition of MHC isoforms were analyzed using Image J software (version 1.52a, NIH) and assessed by counting the number of specific muscle-fibers, type I (blue), type IIa (green), and type IIx/b (black).

### Western blot analysis

SOL muscles were homogenized (1:10 w/v) in T-PER^®^ buffer (#78510, ThermoFisher Scientific) containing a Halt™ Protease and Phosphatase Inhibitor Cocktail (#78446, ThermoFisher Scientific), using a Polytron™ PT 2500E Homogenizer (#08-451-320, Kinematica). The homogenates were centrifuged at 10,000 × g for 15 min at 4˚C for total protein extraction. Protein concentration was assessed using a Pierce™ Coomassie Plus Assay Kit (#23236, ThermoFisher Scientific). Aliquots of the supernatant were heated in 4X Bolt™ LDS Sample Buffer (#B0008, Invitrogen) at 70˚C for 10 min and later cooled on ice for 10 min. An equal amount of 40 μg protein was loaded onto the Bolt™ 4-12% Bis-Tris Plus Gels (#NW-04125BOX, Invitrogen) for 1 hour at RT in Bolt™ MOPS Running Buffer (#B0001-02, Invitrogen) and then transferred to Bolt™ Transfer Buffer (#B00061, Invitrogen) using 0.2 μm PVDF membranes (#88520, ThermoFisher Scientific) for 1 hour at 4˚C. Non-specific proteins were blocked for 60 min at RT in 5 % bovine serum albumin (BSA) with Tris-buffered saline containing 0.1% tween-20 (TBS-T), and the membranes were incubated with the designated primary antibodies for 24 hours at 4˚C. The antibodies used are as follows: PAX7(#81975), MYOD(#71629), MYOG(#12732), TFAM(#376672), MAFbx (#166806), and MuRF-1 (#398608) from Santa Cruz Biotechnology; MitoProfile®total OXPHOS(# 110413) and Cathepsin L(#133641) from Abcam; α-ACTN(#6487), BECLIN-1(#3738), FOXO3α(#12829), p-FOXO3α^Ser253^(#13129), LC3(#12741), and p62(#5114) from Cell Signaling Technology; LAMP-2(#PA1-655) from ThermoFisher Scientific. After overnight incubation, the membranes were washed with 1X TBS-T (3 × 10 min) and further incubated with the designated secondary antibodies conjugated with HRP for 1 hour at RT: goat anti-rabbit (#1148960), rabbit anti-goat (#811620) from ThermoFisher Scientific; goat anti-mouse (#2005) from Santa Cruz Biotechnology. Then, the membranes were washed with 1X TBS-T (3 × 10 min). Immunoreactive proteins were detected using a SuperSignal™ West Dura Extended Duration Substrate (#34076, ThermoFisher Scientific). The images for visualization were acquired by ChemiDoc™ MP Imaging System (#12003154, Bio-Rad), and band intensities were quantified using Image Lab™ software version 5.2.1 (#1709690, Bio-Rad). The protein expression was normalized with total proteins stained with Ponceau solution (#P7170, Sigma-Aldrich). All protein expression levels were presented as a fold change by percentage.

### Statistical analysis

All data were analyzed using the SPSS 25.0 software package (Chicago, IL, USA). Values are presented as means ± SEM. Comparisons between groups for each dependent variable were conducted using a one-way ANOVA, and Tukey’s honestly significantly different (HSD) test was adopted for post-hoc analyses. Statistical significance levels were set at α < 0.05.

## RESULTS

### Chronic DOX treatment induces severe body weight reduction, but not skeletal muscle atrophy

**Figure 2.**
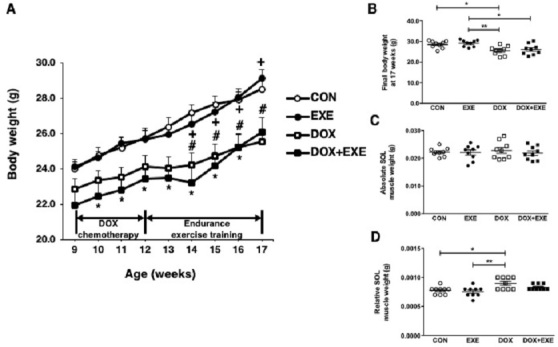
Effects of the endurance exercise training after DOX chemotherapy on (A) body weight, (B) final body weight at 17 weeks, absolute soleus muscle weight, and (D) relative soleus muscle weight. Sedentary control group; CON, Endurance training group; EXE, Doxorubicin chemotherapy group; DOX, Doxorubicin chemotherapy, and endurance exercise training group; DOX+EXE. n=9 animals per group. An asterisk for (A) denoted significant differences; both CON and EXE vs. DOX+EXE (*), CON vs. DOX (#), EXE vs. DOX-+EXE (+). (B and D) Values are means±SEM. Statistical significance was evaluated using one-way ANOVA.

Distinguishable chronological body weight change patterns were observed between the non-chemotherapy (CON and EXE) and chemotherapy (DOX and DOX + EXE) groups (Fig. 2A). Chronic DOX treatment significantly decreased the final body weight in DOX, compared with CON and EXE (Fig. 2B). Interestingly, there was no wasting of the absolute SOL muscle weight in each group (Fig. 2C). Additionally, the relative SOL muscle weight was significantly increased by DOX treatment, compared with CON and EXE (Fig. 2D).

### DOX chemotherapy is myotoxic and impacts specific muscle-fiber types

**Figure 3.**
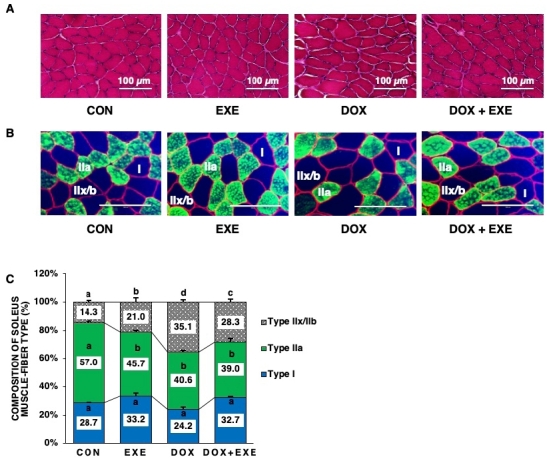
Effects of endurance exercise training after DOX chemotherapy on histological phenotype in soleus muscle. (A) H&E staining for each group, (B) Skeletal muscle-fibers typing by MHC isoforms (Type I; blue, Type IIa; green, Type IIx/IIb; black, dystrophin; red) for each group, and (C) Composition of soleus muscle-fiber type for each group. Sedentary control group; CON, Endurance training group; EXE, Doxorubicin chemotherapy group; DOX, Doxorubicin chemotherapy and endurance exercise training group; DOX+EXE. Values are means±SEM. Statistical significance was evaluated using one-way ANOVA. Different letters (a, b, c, and d) represent significant differences at p < 0.05.

The effects of DOX exposure on myotoxicity and muscle fibers were examined. DOX chemotherapy induced abnormal features, with irregular myofiber size and centronucleation dislocation. These pathological characteristics were reversed by EXE (Fig. 3A). Moreover, the MHC type IIa isoform composition of the SOL muscle-fiber significantly decreased and type I composition reduced in response to DOX treatment. However, EXE training-induced adaptation allowed favorable shifting of muscle-fiber types to oxidative metabolism, with an increase in type I and decrease in type IIa (Fig. 3B and 3C).

### Endurance exercise contributes to myofiber stabilization and mitochondrial function-related protein expression, but parallels myogenesis against chronic DOX-induced myotoxicity

**Figure 4.**
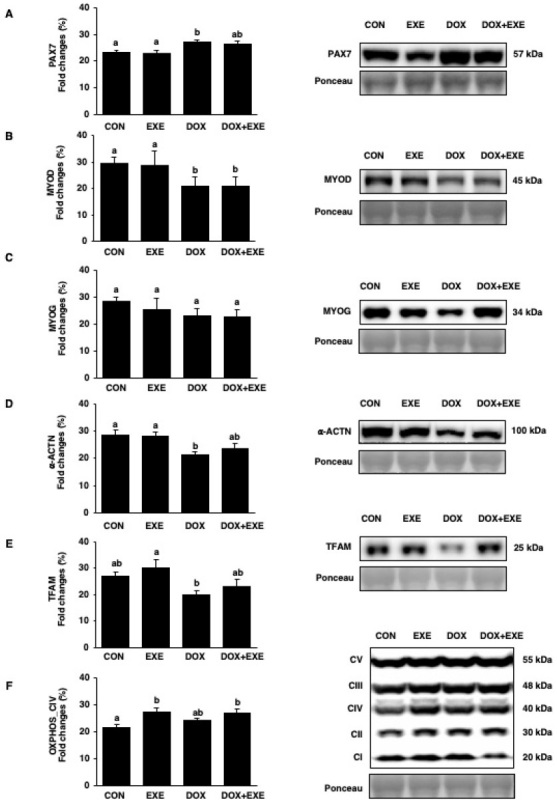
Effects of the endurance exercise training after DOX chemotherapy on satellite cells activation, myofiber formation, and mitochondrial function in soleus muscle. Protein expression levels of (A) PAX7, (B) MYOD, (C) MYOG, (D) α-ACTN, (E) TFAM, and (F) OXPHOS_CIV. All membranes were normalized by Ponceau staining. Sedentary control group; CON, Endurance training group; EXE, Doxorubicin chemotherapy group; DOX, Doxorubicin chemotherapy and endurance exercise training group; DOX+EXE. Values are means±SEM. Statistical significance was evaluated using one-way ANOVA. Different letters (a and b) represent significant differences at p < 0.05.

The ability of EXE training to mitigate DOX chemotherapy-induced myotoxicity through the potential regeneration capacity of skeletal muscle was investigated. The levels of PAX7 protein, a quiescent to proliferative satellite cell activation marker, were significantly upregulated in DOX and DOX+EXE, compared to CON and EXE (Fig. 4A). The expression of MYOD protein, a differentiation marker of myogenic regulation, was significantly diminished in DOX and DOX+EXE (Fig. 4B), while the levels of a myofiber formation (myogenesis) marker, MYOG protein, were not changed among the groups (Fig. 4C). Moreover, the Z-line expression of α-ACTN protein, a supporting structural myofiber protein, was inhibited in DOX, but the expression was restored in DOX+EXE (Fig. 4D). Since DOX disrupts mitochondrial function, this study examined whether EXE could rescue it. The protein levels of mitochondrial transcription factor A (TFAM) were significantly inhibited by DOX treatment (Fig. 4E), while oxidative phosphorylation (OXPHOS) complex IV (CIV) was significantly upregulated in response to EXE (Fig. 4F).

### DOX chemotherapy stimulates FOXO3α activity, but its alteration is not associated with major proteolytic systems in the skeletal muscle

**Figure 5.**
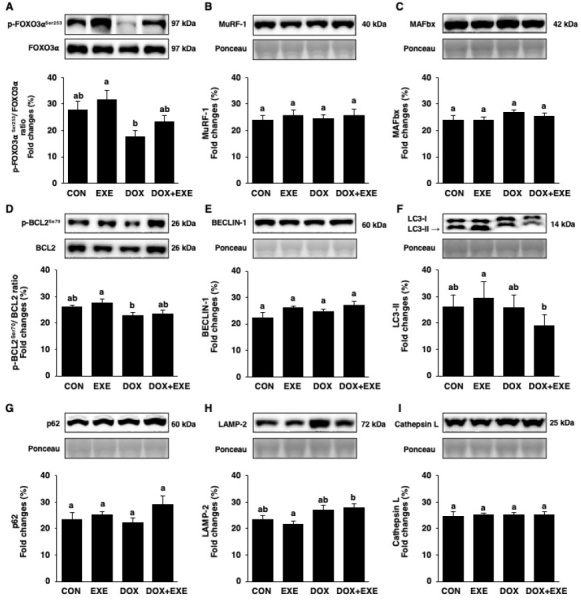
Effects of the endurance exercise training after DOX chemotherapy on FOXO3α, muscle-specific E3 ubiquitin ligases, and autophagy signaling pathways in soleus muscle. Sedentary control group; CON, Endurance training group; EXE, Doxorubicin chemotherapy group; DOX, Doxorubicin chemotherapy and endurance exercise training group; DOX+EXE. Proteins expression levels of (A) p-FOXO3α^Ser253^/FOXO3α ratio, (B) MuRF-1, (C) MAFbx, (D) p-BCL2^Ser70^/BCL2 ratio, (E) BECLIN-1, (F) LC3-II, (G) p62, (H) LAMP-2, and (I) Cathepsin L. All membranes were normalized by Ponceau staining. Values are means±SEM. Statistical significance was evaluated using one-way ANOVA. Different letters (a and b) represent significant differences at p < 0.05.

I wondered whether DOX chemotherapy-induced myotoxicity could be mediated by major catabolic systems such as the ubiquitin-proteasome system (UPS) and autophagy and if EXE postconditioning could buffer the detrimental effects on skeletal muscles. They were regulated by FOXO3α expression. Thus, FOXO3α activity and its downstream signaling pathways, including muscle-specific ubiquitin E3 ligases (MuRF-1 and MAFbx) and autophagy-related proteins, were explored. FOXO3α was activated by decreasing phosphorylation in DOX, compared with EXE, but returned to basal level in DOX-+EXE (Fig. 5A). However, neither MuRF-1 nor MAFbx were significantly changed (Fig. 5B and 5C). Ironically, most of the autophagy-related proteins, including the p-BCL2^Ser70^/BCL2 ratio, BECLIN-1, LC3-II, p62, LAMP-2, and Cathepsin L, did not show significantly overlapping effects (Fig. 5D - 5I).

### Molecular mechanisms of DOX-induced myotoxicity in mice

**Figure 6.**
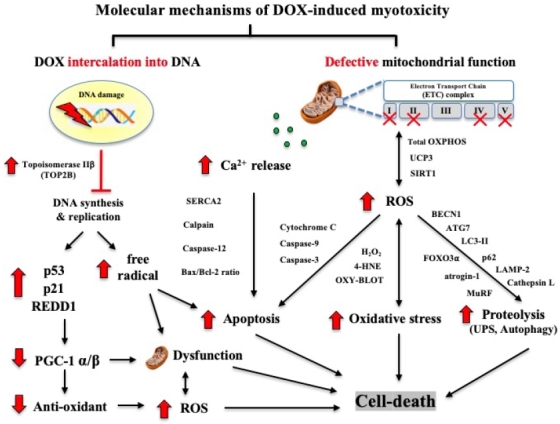
Molecular mechanisms of DOX-induced myotoxicity in mice.

The cellular and molecular mechanisms of the protective effects of EXE training against DOX chemotherapy-induced myotoxicity are summarized in Fig. 6.

## DISCUSSION

The findings of this study showed, for the first time, the protective effects of regular EXE training on the skeletal muscle remodeling against chronic DOX chemotherapy-induced myotoxicity in comparison to previous studies that examined high-dose DOX treatment with single-bout injection^18-20,39,44,48^. The high-dose single bout injection may not be applicable in clinical situations due to the lack of accumulative dosage of the drug. Thus, recent studies have applied repetitive low-dose DOX chemotherapy^12,40,41^. Besides, previous studies designed for DOX treatment and EXE training have been proposed to have positive effects regarding EXE preconditioning ^18-20,39, 44,48,49^. In addition, the timing of EXE training was planned during DOX administration^41^ or before and during DOX injection^43^. Regarding this, a modified experimental design is required for the practical avenue through EXE training after DOX treatment (named EXE postconditioning) in compliance with chemotherapy ^40,42^. Therefore, this study was performed for a repetitive four cycles of DOX chemotherapy, followed by 5 weeks of EXE training, including 1 week of treadmill adaptation.

Several studies report that chronic DOX treatment resulted in a significant decline in body weight. However, a relative muscle weight of SOL was paradoxically increased, whereas an absolute muscle weight of SOL was not affected. Likewise, previous studies emerged with the same patterns for changes in body weight resulting from sub-chronic DOX treatment (2 mg/kg/week for 7 weeks) in young male Sprague-Dawley (SD) rats, but there was no description of the effect on the skeletal muscle mass^43^. Recently, studies using young female ovariectomized SD rats not only indicated that DOX administration (4 mg/kg, biweekly three times injection) caused a loss in body weight but also mentioned no interaction or effects for either an absolute or a relative SOL muscle weight^41^. Currently, there is no clarity regarding DOX treatment-induced reduction in the overall body weight, but not corresponding to muscle weight. However, a possible explanation may be that the ratio of body composition to chronic DOX exposure is strongly impacted by both fat-free mass (skeletal muscle tissue) and fat mass [40]. Yet, the adipose tissues were not measured since the study only focused on the skeletal muscle tissue. Thus, further investigations must inculcate the measurement of both the muscle and fat mass to outline the effect of DOX chemotherapy clearly.

In an attempt to ascertain the relevance of DOX chemotherapy-induced myotoxicity, the pathological features with severely damaged myofibers were examined. Similar data showed that DOX exposure provided SOL muscle with a significantly lessened centronucleation (a marker of muscle-fiber regeneration) in male SD rats^42^. Typically, the composition of the SOL muscle-fiber type by MHC isoforms includes the following; type I (30.6 %), type IIa (49.1 %), and type IIx/b (14.9 %), in C57BL/6 mouse models^50^. The current data also show similar distribution; type I (28.7%), type IIa (57.0 %), and type IIx/b (14.3 %) for CON in the same species of mice SOL muscle. Since DOX-induced contractile impairments have a predominant expression in rat SOL muscles^22^, histological phenotype indicates that the transformation of muscle-fiber types occurs from fast-twitch (type IIa + type IIx/b) to slow-twitch (type I) in response to EXE in this study. Thus, EXE training contributes to reconstruction against the chronic DOX-mediated myotoxicity in a specific muscle fiber type-dependent manner.

To investigate the potential mechanisms for the protective effects of EXE training against DOX-induced myotoxicity, this study focused on the muscle regeneration capacity through satellite cell activation, including myogenic regulatory factors (MRFs) and the structural protein of myofibers. First, PAX7 is known as a marker for identifying quiescent and satellite cell activation^51^. It also plays a role in maintaining the proliferation of progenitors and preventing early myogenic differentiation^52,53^. Second, MYOD is a helix-loop-helix transcription factor that plays a major role in regulating myogenic differentiation as one of the MRF family members^32,33^. The current results showed that DOX chemotherapy upregulates PAX7 protein expression levels, whereas it downregulates MYOD expression. Hence, it may be a compensatory reaction against chronic DOX-induced myotoxicity. Recently, a study using cancer-induced cachexia animal models showed PAX7 upregulation compared to controls, and voluntary wheel running of physical activity counteracted with PAX7 overexpression^54^. Moreover, the data for MYOD protein expression are consistent with in vitro studies, as DOX treatment inhibited it in mouse embryonic fibroblast cells and C2 myoblast cells^32,33^. This result is contrary to an in vivo study that showed MYOD stimulation by an acute DOX injection within the SOL muscle in SD male rats^44^. Third, MYOG is a transcription factor that is involved in the MRF family members^55^, and an important protein that engages in differentiation during myogenesis, fusion into myotube, and maturation into myofiber^31^. MYOG has been well-described for muscle regeneration and improvement of exercise capacity in adult MYOG deficient mdx mice^56^. However, these data unexpectedly show that MYOG activity was not sufficient to be regulated in response to the drug and exercise interventions. Lastly, α-actinin (α-ACTN) is a structure-fortifying protein in myotube, which is involved in the Z-lines construction with an attachment for actin filaments that contributes to maintaining stability during muscle contraction^57^. This study reveals that α-ACTN protein levels were suppressed by chronic DOX exposure, but EXE training improved the α-ACTN levels. Thus, α-ACTN may be a prime contributing factor that mitigates against DOX-induced myotoxicity.

FOXO3α is the forkhead box class O (FOXO) family of transcription factors, and its dynamic activation is involved in numerous muscular atrophy^20^ since they promote the expressions of key enzymes linked to the ubiquitin-proteasome system (UPS) and autophagy^18,58^. Specifically, the FOXO3α upregulates the muscle-specific E3 ubiquitin ligases such as muscle ring finger-1 (MuRF-1) and muscle atrophy F-box (MAFbx) leading to polyubiquitination of the target muscle proteins for degradation^59,60^. A recent study has demonstrated that acute DOX exposure upregulated the MuRF-1 and MAFbx mRNA levels in adult male SD rats using SOL muscles^20^. Contrarily, DOX chemotherapy did not affect both the MuRF-1 and MAFbx protein levels, despite FOXO3α activation, in this study. Thus, there is a difference in detecting the location of target proteins between the transcription and translation levels. Indeed, FOXO3α activity is dependent on its phosphorylation status for detection, either in the nucleus or cytoplasm^61^. In addition, emerging reports shows prospect for the FOXO family of signaling and their function as therapeutic targets in cancer^62,63^. Hence, this study suggests the inhibition of FOXO3α as a potent therapy not only for DOX-induced myotoxicity but also for cancer cachexia-induced muscle atrophy.

Autophagy is a major proteolytic system that uses the lysosomal proteases to eliminate damaged/dysfunctional proteins and various cellular components to maintain cellular homeostasis^59,64^ however, dysregulation of autophagy results in skeletal muscle disease^18,19,58,64,65^. Acute DOX treatment-induced excessive autophagy is strongly associated with myotoxicity in male SD rats, but preconditioning with short-term EXE provides protective effects to SOL muscles^18^. In contrast, neither chronic DOX nor DOX+EXE exhibits autophagy dysregulation. Therefore, it can be presumed that the skeletal muscle tissue exposed to acute DOX treatment may seem to be upregulated for autophagy spontaneously due to a strong dosage. Nonetheless, the muscle tissue exposed to chronic DOX exposure might not affect autophagy levels without further exacerbation. Supporting this, a recent study demonstrated that chronic DOX treatments do not alter autophagy in SOL muscles of ovariectomized female SD rats^41^. There are some limitations to this study. Although the p-mTOR^Ser2448^/ mTOR ratio was analyzed to observe the anabolic pathways, there were no significant differences among groups. Unfortunately, the lysate volume of the SOL muscles for the WB assay became exhausted during the analysis for the p-AKT^Ser473,Thr308^/AKT ratio to evaluate the balance of protein-turnover.

In conclusion, this study suggests that EXE postconditioning plays an essential role in the restoration of muscle quality and maintenance for the inherent traits in slow-twitch muscles against chronic DOX chemotherapy-induced myotoxicity. Thus, regular EXE training after cancer chemotherapy should be recommended as a therapeutic strategy for cancer survivors to alleviate chemotherapy-mediated myotoxicity.

